# Low‐Cost Ambient Pressure Drying Approach for Highly Porous Nanomaterial Structures

**DOI:** 10.1002/smtd.202502308

**Published:** 2026-04-20

**Authors:** Erik Greve, Pia Pooker, Jan‐Ole Stern, Rainer Adelung, Valeria Nicolosi, Dahnan Spurling

**Affiliations:** ^1^ Functional Nanomaterials Institute For Materials Science Faculty of Engineering Kiel University Kiel Germany; ^2^ Centre for Research on Adaptive Nanostructures and Nanodevices (CRANN) & Advanced Materials Bio‐Engineering Research Centre (AMBER) Trinity College Dublin, School of Chemistry Dublin Ireland

**Keywords:** 2D materials, aerogel, ambient drying, critical point dryer, drying mechanisms, nanomaterials, porous materials

## Abstract

Drying highly porous structures presents a fundamental challenge, as capillary forces during evaporation collapse pores, limiting utility. Despite advances in sol–gel processing and nanostructure synthesis, techniques such as critical point drying (CPD) drive up cost, limit size and reduce throughput. This creates a major bottleneck for scalable fabrication of aerogels, membranes, and advanced lightweight materials. Here, a simple, low‐cost, solvent‐based technique for ambient temperature and pressure drying of highly porous graphene, MXene, PEDOT:PSS, MoS_2_, and TiO_2_ structures is reported. The effectiveness of low surface tension solvents, hexamethyldisilizane and tetramethylsilane, are compared as drying media, while measurements of mass and volume during drying provide insight into the process. Using HMDS, capillary forces are minimized, and the microstructure of the materials is preserved. For example, shrinkage as low as 1.3% for graphene at an ultralow density of 0.018 g cm^−3^ was achieved. TMS showed inferior drying performance in direct comparison to HMDS due to its high vapor pressure and rapid evaporation. The use of low surface tension solvents also allowed the creation of large 11 × 11 cm graphene structures, greatly exceeding the chamber size of typical CPD equipment. Finally, a comparison of the cost of ambient drying and CPD is presented.

## Graphical abstract

We propose a simple, low‐cost method to dry highly porous nanomaterial structures at ambient temperature and pressure. Using low surface tension solvents, this technique minimizes capillary forces, preventing pore collapse and material shrinkage. This bypasses the need for expensive and size‐limiting critical point drying, enabling scalable production of large, high‐quality, low‐density structures.

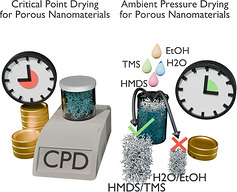

## 1 Introduction

Porous materials, characterized by their interconnected voids, offer a unique balance of low density, high surface area, and tunable properties, making them essential for applications including energy storage, catalysis, biomedicine, insulation, and filtration systems [1]. They are characterized by their pore size *d* (microporous: *d* < 2 nm, mesoporous: 2 nm < *d* < 50 nm, macroporous: *d* >50 nm), the porosity *ϕ*, the pore shape, and accessibility [2]. Notable examples include zeolites, metal organic frameworks (MOFs), metal foams, aerogels, and an emerging class known as framework aeromaterials (FAMs), first created by Mecklenburg et al. [3]. using a CVD process and later synthesized by Rasch et al. using a wet chemical approach [4] These porous materials are fabricated by coating a sacrificial template of tetrapodal zinc oxide (t‐ZnO) with a nanomaterial and subsequently removing the template. Wet chemical coating can be achieved either via dropcasting or wet chemical synthesis. This process is highly versatile, enabling the creation of FAMs from materials such as graphene [3], PEDOT:PSS [5], MXenes [6], titanium dioxide (TiO_2_), and molybdenum disulfide (MoS_2_)—all of which are explored in this study. Like many other highly porous materials, drying FAMs is a complex task as it involves preserving the delicate nano/microstructure during solvent removal. The drying procedure typically used for aerogels or FAMs is critical point drying (CPD), which is not only expensive but also time‐consuming [7, 8, 9]. It requires specialized equipment, liquid CO_2_, and ultra‐high purity ethanol, while critical point driers (CPDs) themselves generally have a small chamber (no more than several cm), which severely limits final size and shape [2, 9, 10]. All of these restrictions make it not only challenging but the most expensive step in aerogel production [7, 8, 9, 10].

To overcome the limitations of conventional drying methods, we propose the use of readily available, low surface tension solvents as drying media. These can not only achieve similar material quality as CPD, but also larger sizes—all while reducing the time, cost, and complexity of preparing highly porous nanomaterial structures [7, 8, 9, 10].In this study, two low‐surface‐tension solvents—hexamethyldisilazane (HMDS) and tetramethylsilane (TMS)—were evaluated for their suitability as ambient drying agents for FAMs made from a wide range of nanomaterials. Both have been previously used for ambient drying of animal and plant tissues as an alternative to CPD or freeze drying [8, 9, 11, 12]. Despite promising results in preserving organic structures, their use as a drying medium in highly porous inorganic systems remains unexplored until now.

**FIGURE 1:**
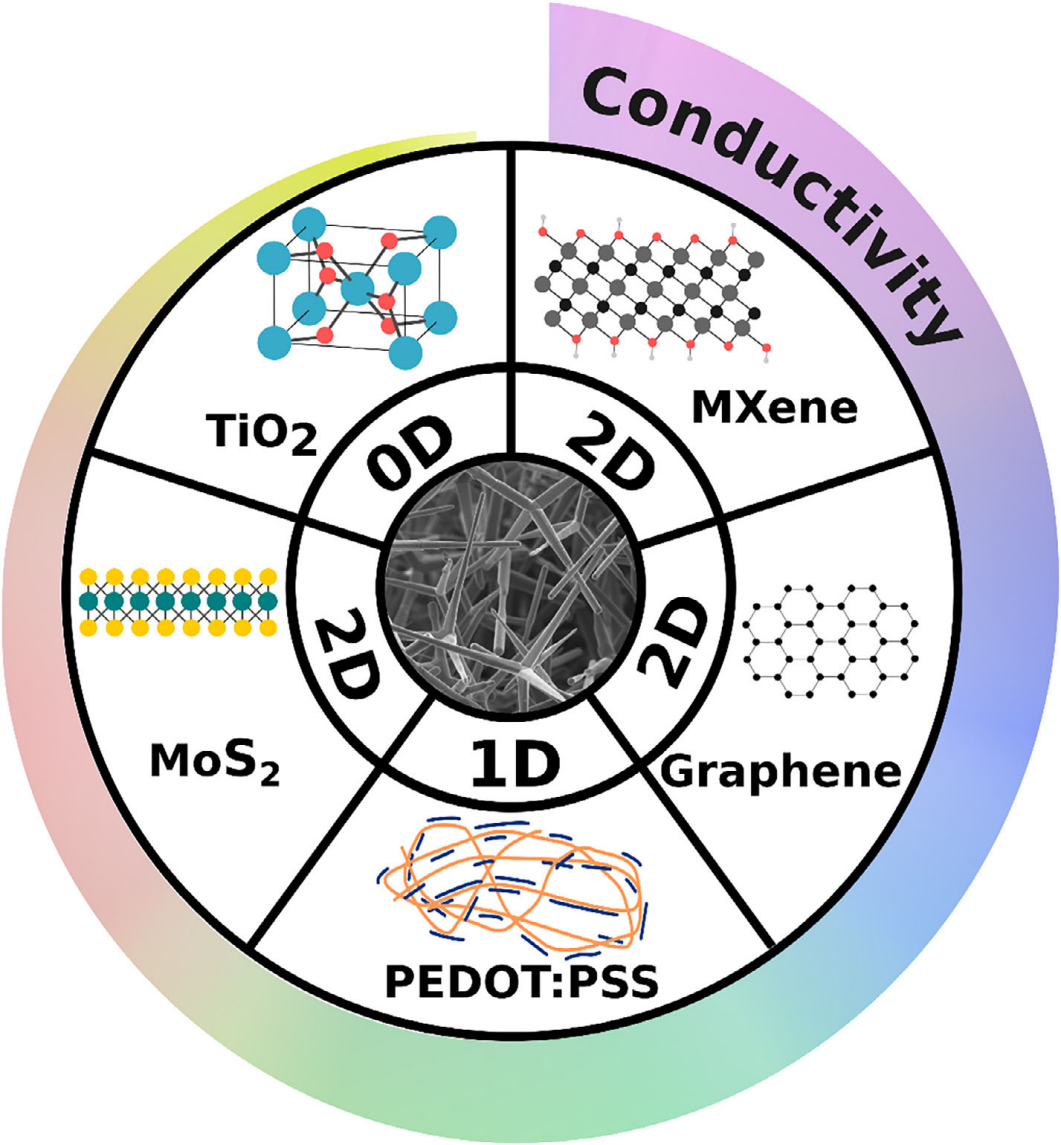
Overview of the nanomaterials investigated in this study, roughly ranked by decreasing electrical conductivity. The visualization incorporates material dimensionality and crystal structure, with relative conductivity differences represented through color gradients and concentric arrangement. Figures were partially adapted from references (TiO_2,_ MXene, MoS_2_) [35, 36, 37].

To highlight the versatility of the proposed drying procedure, FAMs were created from different nanomaterials (see Figure 1), which differ significantly from one another in their physical and chemical properties. Graphene, the archetypal 2D material, revolutionized materials science in 2004 with its exceptional mechanical strength (Elastic modulus ∼800–1000 GPa [13, 14]) and high electrical conductivity, prompting extensive investigation into its potential [15, 16]. Following this, in 2011, MXenes emerged as an exciting family of 2D transition metal carbides and nitrides [17]. These materials, derived from MAX phases, offer tunable properties due to their metallic nature and diverse surface terminations and exhibit high elastic moduli of approximately 215–480 GPa [18, 19], making them highly attractive for electronics, energy storage, and catalysis [20, 21]. Molybdenum disulfide (MoS_2_) is another prominent 2D material and member of the transition metal dichalcogenides (TMDs). These materials are characterized by their mechanical flexibility, tunable band gaps, and distinct surface interactions, allowing for their easy exfoliation into ultrathin 2D sheets [22]. MoS_2_ has an elastic modulus in the range of 200 to 400 GPa [23]. Beyond these 2D materials, PEDOT:PSS stands out as a useful conductive polymer [24, 25]. With a comparatively low elastic modulus of only 0.05–3 GPa [26, 27], PEDOT:PSS combines high electrical conductivity, optical transparency, and flexibility. These properties enable applications in biomedicine and solar cells [28, 29, 30]. Rounding out this diverse group, titanium dioxide (TiO_2_) is a well‐researched semiconductor primarily valued for its photocatalytic properties [31]. Depending on phase and morphology, TiO_2_ thin films exhibit elastic moduli in the range of 60–157 GPa [32, 33], and can be synthesized into high‐surface‐area aerogels that are particularly attractive for environmental and energy‐related applications [31, 34]. All these materials can benefit from being utilized in a 3D porous architecture like FAMs as they offer high accessible area for surface‐controlled processes.

This work aims to expand the understanding of ambient condition drying of porous networks, with a focus on the correlation between solvent properties and structural preservation.

## 2 Experimental Methods

In this study, 96% ethyl alcohol (EtOH), 99% EtOH, HMDS, TMS, and isopropanol were obtained from Carl Roth. The aqueous exfoliated graphene (EG) dispersion was purchased from Sixonia Tech at a concentration of 2.8 mg ml^−1^. It is diluted to 1.4 mg ml^−1^ and sonicated for 10 min (30% amplitude, 3 s on, 3 s off) using an ultrasonic probe (Bandelin Sonoplus UH400). PEDOT:PSS was sourced from Sigma Aldrich and mixed into an aqueous dispersion according to Barg et al. using DVS as a crosslinking agent [5]. The aqueous MXene dispersion was produced according to a procedure reported previously by Spurling et al. [6]. It was diluted to a concentration of 2 mg ml^−1^.

For TMDs, using MoS_2_ as an example, 1.6 g of MoS_2_ powder was added to 80 mL of isopropanol (IPA). The dispersion as sonicated using an ultrasonic probe (60% amplitude, 6 s on, 2 s off). Then, the dispersion was centrifuged at 10 000 rpm to obtain the MoS_2_ nanoflakes. These were redispersed in IPA through bath sonication. Afterwards, they were then centrifuged at different speeds (1000, 3000, and 5000 rpm) for 1 h and the supernatant was retained (size selection of MoS_2_). The dispersion was then diluted to a concentration of 1.5 mg ml^−1^ and mixed with an EG dispersion of 1.5 mg ml^−1^ in a ratio of 2:1 to ensure stability of the final FAM.

### 2.1 FAM‐Preparation

Tetrapodal zinc oxide (t‐ZnO) microparticles were synthesized using the flame‐transport method [38]. To create templates from the microparticle powder, the required amount (0.3 g cm^−3^) of powder is weighed out and compacted into the desired shape (12 mm × 2 mm). After pressing, these structures are sintered (1150°C, 5 h), allowing the tetrapod arms to fuse together and form a stable, oxygen vacancy‐rich hydrophilic network. Except for TiO_2_‐FAM, the template is subsequently coated with a dispersion of the respective material. Before coating, the respective nanoparticle dispersion is sonicated using an ultrasonic probe to ensure homogeneity. During infiltration, the dispersed particles adhere to the tetrapod arms as the solvent evaporates. This process is repeated several times with drying steps between each infiltration until the 3D network is sufficiently coated (MXene‐FAM: 10 infiltrations, EG‐FAM: 6 infiltrations, PEDOT‐FAM: 10 infiltrations, TMD‐FAM: 12 infiltrations) [4]. For the synthesis of TiO_2_‐FAM, a t‐ZnO template is kept in an aqueous solution of 0.35 M boric acid and 0.15 M ammonium hexafluorotitanate (AHFT) for 72 h [39].

After the respective coating process is complete, the template is completely etched via a wet‐chemical process using acidic solutions (1 M hydrochloric acid, or 25% acetic acid), resulting in a network of interconnected hollow microtubes. This process has been reported previously [4]. The samples are then dried using the protocols described below.

### 2.2 Critical Point Drying

The etched FAM samples were washed with 96% EtOH (5 mL) until the pH was neutral. The samples were then washed two times with 99% EtOH (5 mL) before being transferred to the critical point dryer (Leica EM CPD300).

### 2.3 Ambient Drying

The etched FAM samples were washed with the desired solvent according to the following protocol. For H_2_O, the samples were washed with deionized water (5 mL) until the pH was neutral. The samples were then washed 2 more times with distilled water (5 mL) before removing it from the liquid and placing them on a hydrophobic membrane (Celgard 2500) for drying. For EtOH, the samples were washed according to the protocol for CPD, before placing on a plate for drying. For HMDS and TMS, the samples were first washed with 96% EtOH (5 mL) until the pH was neutral, followed by washing with a 50/50 mixture of 99% EtOH and either HMDS or TMS (5 mL total), respectively. Finally, the samples were washed 2 more times with either pure HMDS or TMS (3 mL) before placing them on a plate for drying. As the FAM is fully wetted and close to 99% porous, the residual volume of solvent in the to be dried FAM is equal to the sample volume (∼0.226 mL).

### 2.4 Monitoring Shrinkage

**FIGURE 2:**
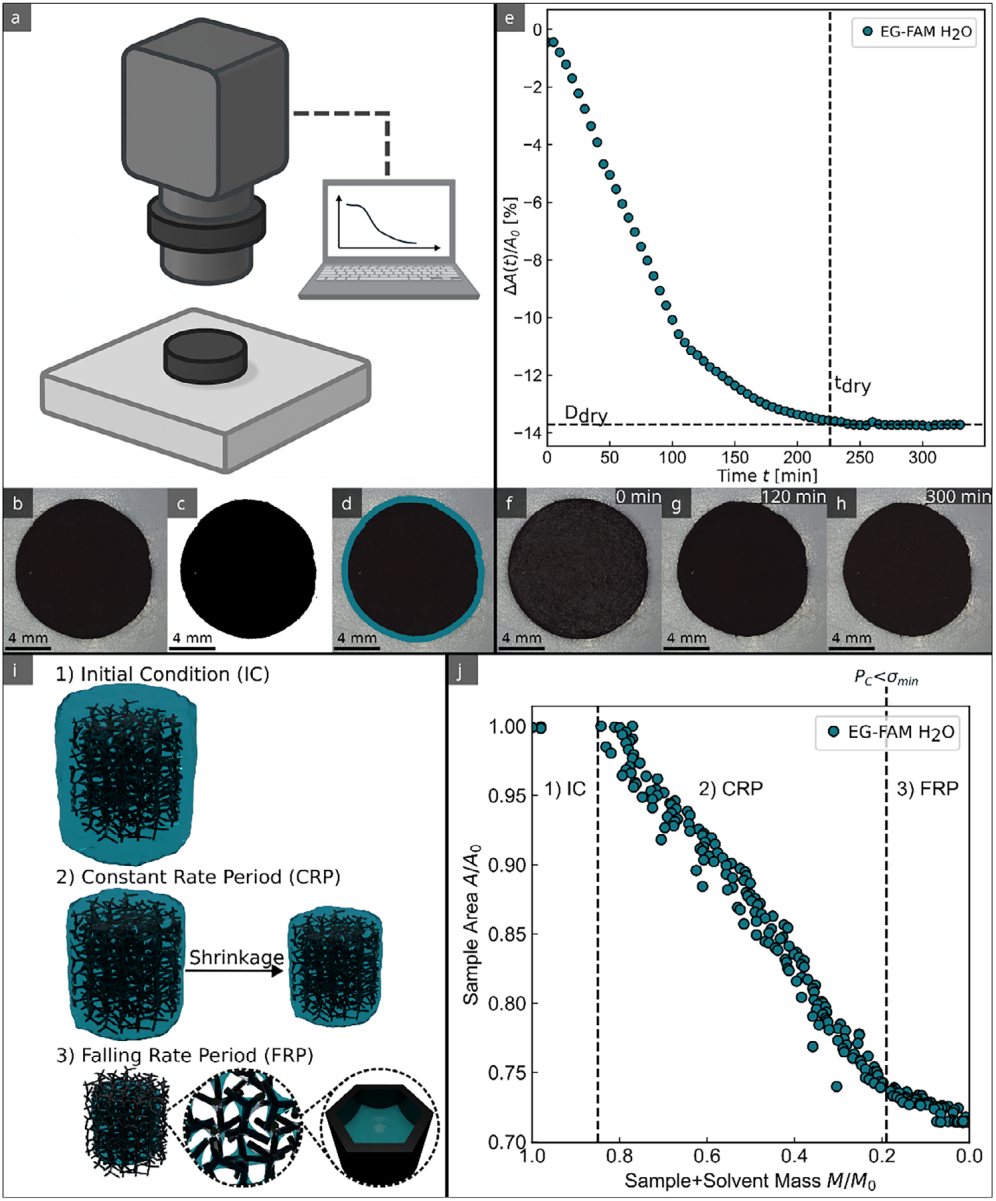
(a) Schematic representation of the camera setup. The sequence from (b) to (d) illustrates the image processing workflow used to identify the sample's contour for subsequent calculation of the enclosed area. (b) Example raw image of the drying sample. (d) Binary (black and white) representation of the grayscale image. (d) Original image with the extracted contour obtained from the binary image superimposed. (e) Shrinkage of an EG‐FAM sample during drying in water. Teal markers show percentage deformation (%) versus time (min); the horizontal dashed line marks the final shrinkage (*D_dry_* = –13.7%), and the vertical dashed line indicates the drying time (*t_dry_* = 226 min when 99% of *D_dry_* is reached) (f–h) Photographs of the same EG‐FAM sample at *t* = 0, 120, and 300 min, respectively. (i) Schematic visualization of the drying regimes. (j) Normalized sample volume *A/A0* versus normalized total mass *M/M_0_*. Vertical dashed lines mark the transitions between three drying regimes: (1) Initial condition: excess solvent evaporates (2) Constant Rate Period: menisci form at the network surface, inducing deformation as solvent continues to evaporate, and the sample yields due to capillary pressure. (3) Falling rate period: Sample stiffened, capillary pressure is not sufficient anymore to deform the sample, the menisci recede into the sample (b) adapted from sol–gel science: the physics and chemistry of sol–gel processing [41].

For each material/solvent combination, three independent samples were imaged every minute during ambient drying using a digital microscope under consistent lighting on a high‐contrast substrate. A schematic of the image acquisition setup is shown in Figure 2a. An original color image is shown in Figure 2b, which is subsequently converted to a gray‐scale image. With the help of Otsu's threshold algorithm, the gray‐scale image is binarized as shown in Figure 2c [40]. The resulting sample contour is shown in Figure 2d, and its enclosed area is used for further analysis of the drying processes.

### 2.5 Mechanical Characterization

Uniaxial compression tests were performed on cylindrical EG‐FAM and PEDOT:PSS‐FAM specimens (height = diameter = 6 mm, 6 and 8 infiltrations, respectively), following critical point drying, using a rheometer (MCR 702e MultiDrive, Anton Paar) at a crosshead speed of 0.3 mm s^−1^. The samples were compressed to half their original height.

### 2.6 Elemental Analysis

Energy‐dispersive X‐ray spectroscopy (EDS) was performed on the dried samples using a scanning electron microscope (Ultra Plus, Zeiss) equipped with an EDS detector (Ultim Max 65, Oxford Instruments).

### 2.7 Economic Considerations

To create the table and data for economic comparison of the different approaches, a minimum 1 dm^3^ container was considered, and the prices were taken from the suppliers listed in Section 2.

## 3 Results and Discussion

### 3.1 Drying Mechanism

To quantify how the samples respond to solvent loss, the area was monitored over time while drying. The deformation *D* is defined as follows:

(1) $$D\left(t\right)=\frac{A\left(t\right)-A_{0}}{A_{0}}\cdot 100\;\%$$

where *A*(*t*) is the time dependent projected sample area, and *A_0_* is the initial sample area. As plotted in Figure 2e for EG‐FAM dried in water, the area initially reduces linearly. After 100 min the deformation rate decreases and after 226 min the shrinkage stops with a total deformation of –13.7% (*D_dry_*). Optical snapshots at selected times (Figure 2f–h) reveal the gradual reduction in the sample area.

In the early drying stage, the area loss is proportional to solvent mass loss (Figure 2j‐2), indicating the constant‐rate period (CRP), during which evaporation proceeds at almost the same rate as for the free liquid [2, 41]. Plotting the area‐loss ratio versus the mass‐loss ratio yields a straight line, showing that volume shrinkage is directly proportional to mass loss of solvent. Due to the initial high mechanical compliance of the material, the capillary pressure is sufficient to keep the network submerged in the solvent, allowing uniform contraction, as illustrated in Figure 2j‐2 [41]. The capillary pressure *P_c_* can be expressed by the Young–Laplace equation as:

(2) $$P_{c}=\frac{2\gamma \cos\theta }{r}$$

where *γ* is the liquid–vapor surface tension, *θ* is the contact angle, and *r* is the radius of the solvent meniscus. This pressure maintains pore saturation and drives uniform shrinkage in the CRP. For a compliant FAM subjected to small pressures and low strains, the response is purely linear elastic, and the modulus remains constant. Once the applied stress exceeds the yield strength, the gel deforms plastically and its resistance to further compression rises as the porosity *ϕ* decreases. In this plastic regime, stress–strain relationship obeys a power‐law dependence on strain, as given by Equation (3) [42].

(3) $$\sigma =P_{0}+\frac{E_{0}}{m}\left({\left(\frac{1}{\varepsilon +1}\right)}^{m}-1\right)$$

where *σ* and *ε* are the stress and strain, *σ_0_* and *E_0_* are the stress and strain at the onset of the plastic regime. The parameter *m* is the power law exponent and describes the strain hardening behavior.

Beyond the CRP (hereafter ∼100 min), the falling‐rate period (FRP) begins. The capillary pressure *P_c_* is no longer sufficient to drive further densification, menisci recede into the pores, and deformation rate slows.

### 3.2 Comparison of Solvents and FAM

**FIGURE 3:**
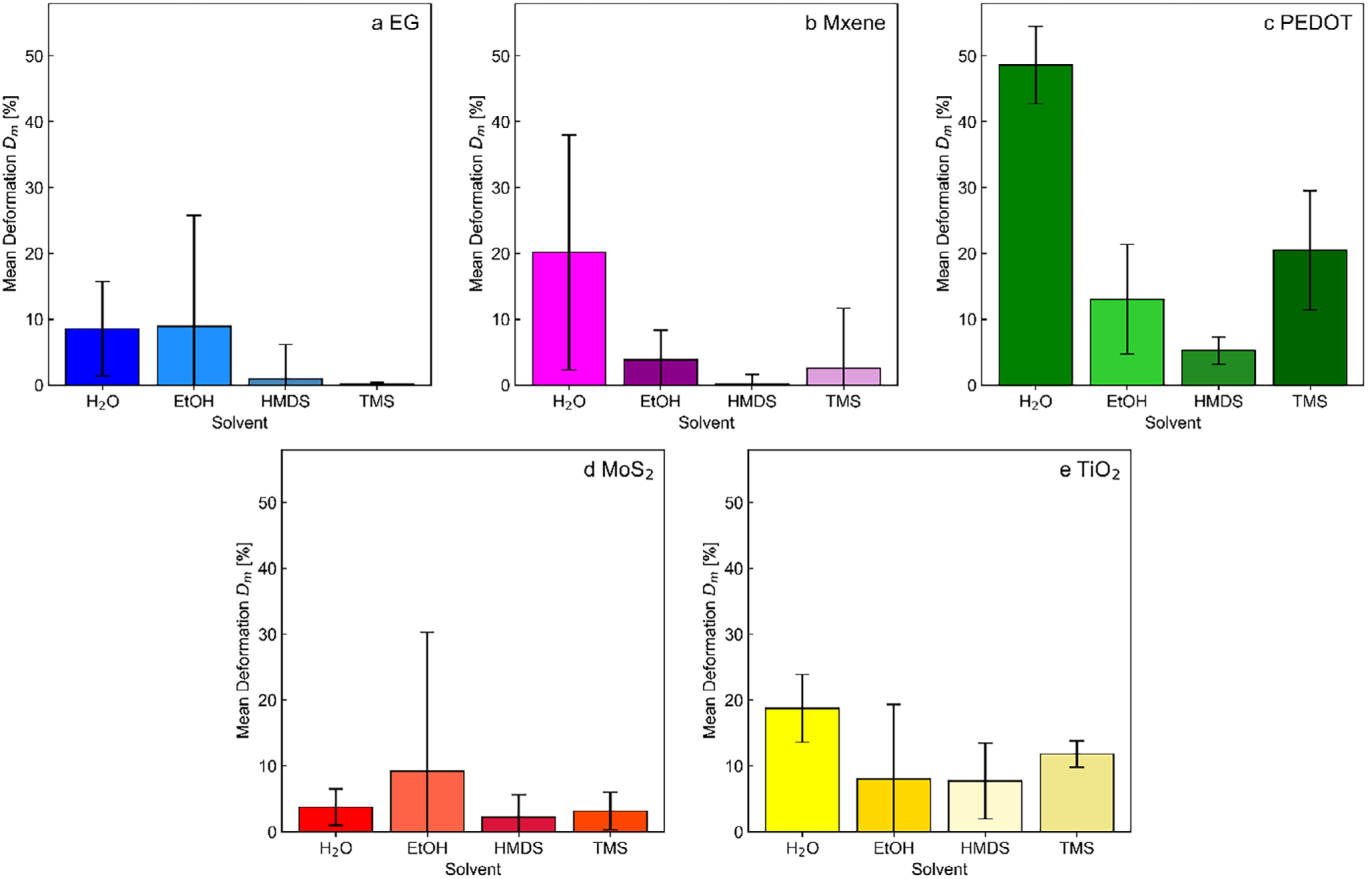
Mean shrinkage of the investigated FAMs dried from different solvents. (a) EG, (b) MXene, (c) PEDOT:PSS, (d) MoS_2_, (e) TiO_2_.

The total deformation of each material dried using different solvents is shown in Figure 3a–e. For all investigated material classes (a EG, b MXene, c PEDOT:PSS, d MoS_2_ and e TiO_2_) drying with water (H_2_O) consistently leads to the highest degree of shrinkage. This pronounced volume reduction is attributed to the high capillary stresses exerted during drying, arising from the inherently high surface tension of water (Table S1), which imposes significant mechanical forces on the porous networks.

Drying porous materials with HMDS results in less shrinkage compared to water or ethanol. This can be attributed to the physical properties of HMDS, which has a surface tension approximately four times lower than that of water (Table S1). Based solely on surface tension, TMS would be expected to induce even less deformation. However, experimental results contradict this assumption, the samples dried with TMS typically exhibit more shrinkage than those treated with HMDS.

Moreover, HMDS can react with the material surface, forming trimethylsilyl groups that reduce interfacial energy and further mitigate shrinkage [2, 43]. Silylation was confirmed by EDS, as the presence of silicon is attributed to the surface modification. The calculated atomic compositions of each FAM dried in HMDS are listed in Table S2. The content of Silicon ranges from 0.03 at % for MXene to 0.66 at % for EG. Si_Kα_‐Lines did not occur in the EDS Spectra of the critical point dried and TMS dried Samples. The corresponding EDS spectra are shown in Figures S1–S3. TMS does not silylate the surface of the FAM, which may account for the greater structural collapse compared to HMDS [2].

**FIGURE 4:**
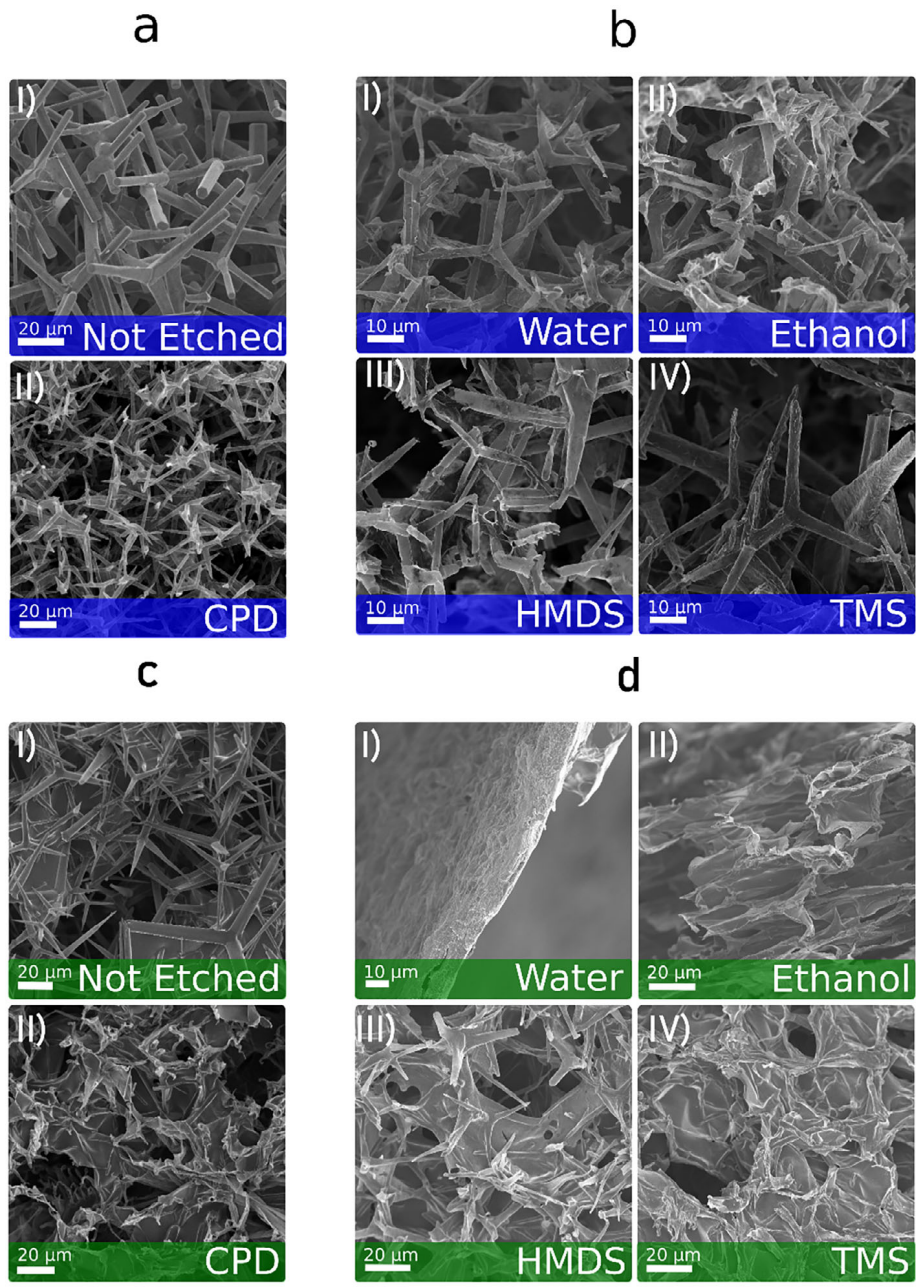
(a) SEM images of the microstructure of EG FAM (a,b) and PEDOT‐FAM (c,d) of a not etched sample (I) and after CPD drying (II). (b) shows the microstructure of a FAM dried in (I) EtOH, (II) H_2_O, (III) HMDS, and (IV) TMS.

Notably, EG‐FAM and MXene‐FAM samples dried with HMDS showed the least shrinkage among all solvent–FAM combinations. This observation is supported by the SEM images shown in Figure 4, which compare EG‐FAM samples in the non‐etched state (Figures 4a‐I), after CPD drying (Figure 4a‐II), and after drying from different solvents, including H_2_O (Figure 4b‐I), Ethanol (Figure 4b‐II), HMDS (Figure 4b‐III), and TMS (Figure 4b‐IV).

In all SEM images, the porous structure and characteristic tetrapodal morphology originating from the sacrificial template remain clearly visible.

In contrast, PEDOT‐FAM deforms the most across all investigated solvents. Being the softest of all materials tested, the capillary pressure can easily deform the whole structure. This is also shown in Figure 4, which compare the microstructure of the non‐etched sample (Figure 4c‐I), the CPD‐dried reference (Figure 4c‐II), and samples dried from H_2_O (Figure 4d‐I), Ethanol (Figure 4d‐II), HMDS (Figure 4d‐III), and TMS (Figure 4d‐IV). The SEM images showing the microstructure of the other FAMs are provided in the SI (Figures S3–S15).

The PEDOT:PSS‐FAM samples dried with water or ethanol collapsed completely, leaving little to no remaining porosity. In contrast, drying with HMDS or TMS also caused significant densification, but to a lesser extent. In these cases, the morphology of the sacrificial template was largely preserved. The stark contrast between EG‐FAM and PEDOT:PSS‐FAM is partially explained by their respective mechanical properties. Figure S16 shows the stress‐strain curve from uniaxial compression tests of both the PEDOT:PSS‐FAM and the EG‐FAM.

For EG‐FAM, the stress–strain curve is linear at small strains, and an apparent yield occurs at 1.7% strain, followed by plastic deformation consistent with Equation (3). By comparison, PEDOT:PSS‐FAM lacks an initial linear region and displays plastic‐like behavior from the start. PEDOT:PSS‐FAM is softer than EG‐FAM until a strain of 25.6%. The attainable strain is governed by the maximum capillary stress, which depends on the material's wetting angle (Equation 2). Notably, PEDOT:PSS is more hydrophilic than graphite (i.e., exhibits a smaller wetting/contact angle), consistent with the observed behavior [44, 45]. These observations support the hypothesis that the final deformation after shrinkage is controlled by both the mechanical properties of the dried material and the material–solvent interaction. In addition, previous work by the Adelung group report mechanical simulations supported by experiments, confirming highly elastic behavior of carbon‐FAMs based on the tetrapodal architecture [3, 46]. This high elasticity explains the observed drying performance of EG‐FAMs, as the occurring capillary stresses are accommodated though elastic, non‐permanent buckling of the hollow EG tetrapods. Due to the structural similarity between EG‐FAM and MXene‐FAM, a similar elastic deformation mechanism can be assumed and thus a similar drying performance is expected.

In the case of TiO_2_‐FAM, however, similar shrinkage across all solvents suggests additional material‐dependent factors. The unexpected behavior of TiO_2_‐FAM may be attributed to its brittle nature and the high porosity of the deposited layer, which likely results in early mechanical failure under drying stress. In contrast to EG‐FAM, buckling of the tetrapodal morphology may not result in an elastic response of the titania thin film, but instead may promote fracture. This may occur before solvent–solid interactions can significantly influence the drying behavior. Raman spectroscopy of TiO_2_‐FAM samples dried from TMS and CPD confirms that no phase transition occurs during solvent treatment (Figure S17).

**FIGURE 5:**
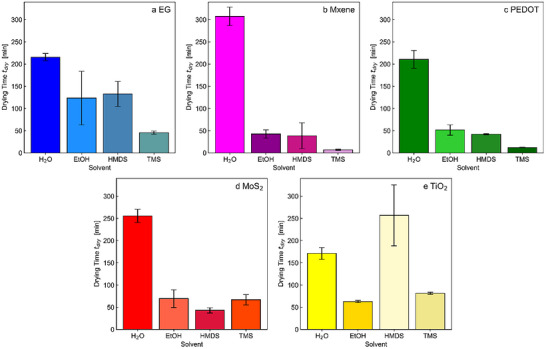
Mean drying time of the investigated porous materials dried from different solvents. (a) EG, (b) MXene, (c) PEDOT, (d) MoS_2_, (e) TiO_2_.

For EG‐, PEDOT:PSS‐, MXene‐, and MoS_2_‐FAMs (Figure 5a–d), the solvent‐dependent drying times follow the same trend: water is slowest, ethanol dries in roughly 50 min, HMDS in about 30 min, and TMS is fastest. Evaporation rate of liquids is proportional to their vapor pressure, but also to their surface tension. Considering the extremely high vapor pressure and low surface tension of TMS the rapid drying times are expected. Comparing the drying times of HMDS and ethanol they appear similar for all FAMs except TiO_2_. Considering the vapor pressure, this is surprising as they differ by a factor of 4, with HMDS having the lower value (see Table S1). However, HMDS possesses a lower surface tension than ethanol, which may explain the similar evaporation rate. Another explanation could be the polarity of each solvent and possible interactions with the substrate leading to a different wetting depending on the FAM, therefore altering drying behavior. TiO_2_‐FAM (Figure 5e) departs from this pattern, as HMDS yields the longest drying time, while ethanol dries the fastest.

**FIGURE 6:**
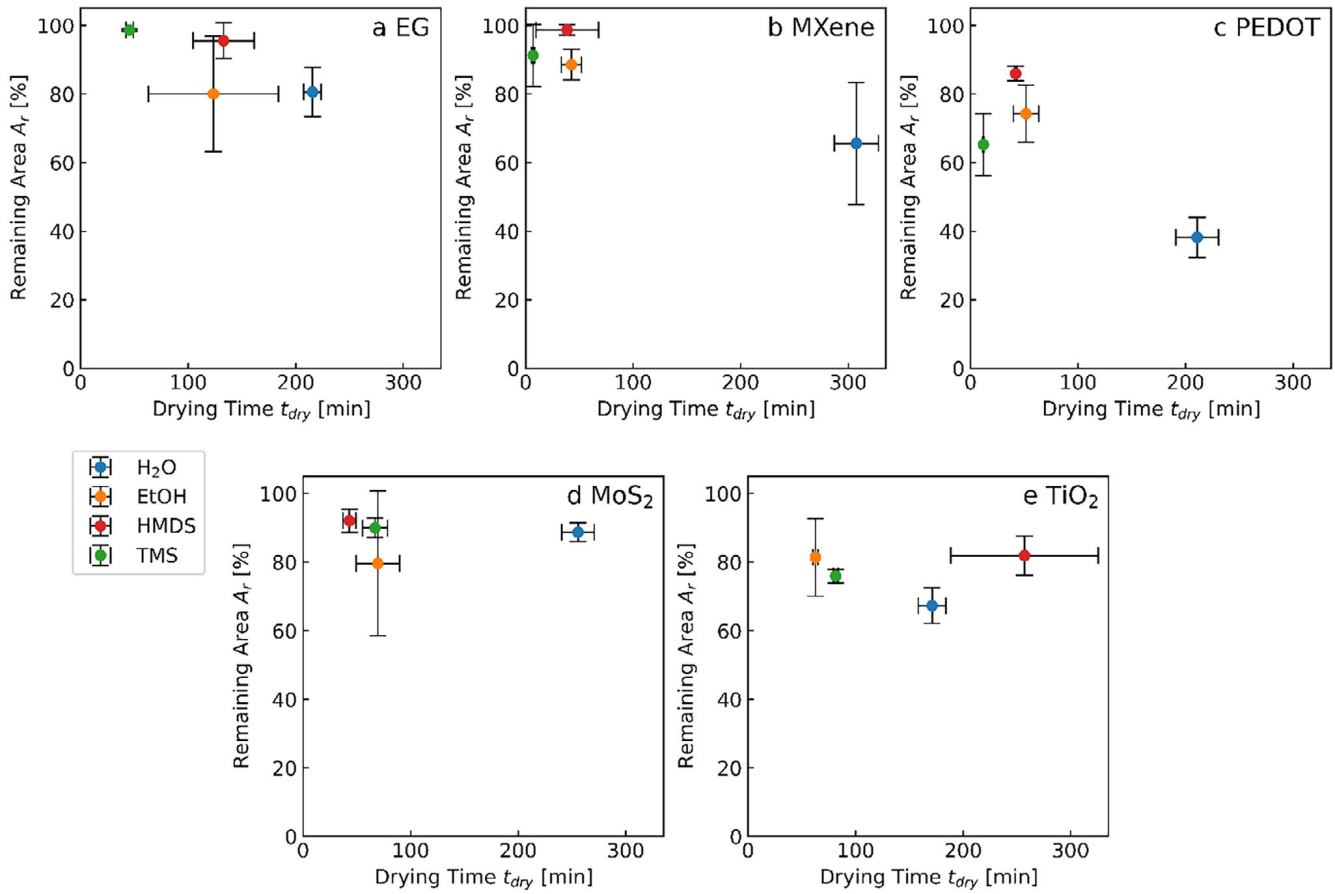
Drying performance of each material (a–e) in different solvents. The *x*‐axis shows drying time, the *y*‐axis shows the remaining area after drying. The error bars correspond to ±1 standard deviation in drying time (horizontal) and remaining area (vertical). The optimal drying conditions correspond to points in the top‐left region of each plot (short drying time, high remaining area).

Figure 6 summarizes for each framework aeromaterial (a EG, b MXene, c PEDOT:PSS, d MoS_2_ and e TiO_2_) the solvent that best balances drying time and structural integrity, by plotting remaining area *A_r_* versus drying time *t_dry_* for each FAM. All the numerical values used to create this graph can be found in Table S3.

Drying behavior of FAMs is governed by solvent properties, solvent‐material interactions, and material properties, especially elastic modulus and yield strength. Across all tested FAMs water causes maximum shrinkage. Except for TiO_2_, all FAMs have minimum shrinkage with HMDS drying. For TiO_2_, ethanol offers the best quality and shortest drying time. Indeed, the method proposed here compares very favorably to other ambient dried aerogel structures, as can be seen in Table S4. Most methods rely on lengthy solvent exchange and surface treatment methods, resulting in poor time efficiency—stretching into days of processing. Adding to this, the EG‐, PEDOT‐, MX‐ and MoS_2_‐FAMS are among the lowest density ambient dried structures (all less than 0.027 g cm^−3^), despite achieving shrinkages of as little as 1.3%, a significant advantage over comparable methods, and achieve indistinguishable results from CPD in these FAMs. This excellent shape retention is crucial for applications that depend on a high porosity and surface fidelity, such as supercapacitor electrodes [47], flow‐through catalysts [48] or actuators driven by rapid air expansion through ultra‐rapid heat transfer [49]. In addition to shape and volume retention, this drying method does not alter or accelerate sample degradation during storage, with samples again indistinguishable from those dried using CPD, as shown in Figure S18, which directly compares the microstructure of EG‐FAM dried with HMDS or TMS immediately after drying and six months of storage.

### 3.3 Economic Calculations and Scalability

Even with the advantages this approach has in terms of time, density, and shrinkage, it would be of limited usefulness were it cost‐prohibitive. However, the process is extremely economical and requires no specialized equipment. CPDs are generally a large capital investment, and ambient drying further eliminates ongoing operational costs such as electricity and maintenance.

A cost breakdown with a detailed representative calculation can be found in Table 1. For solvent cost, we assume an average quantity value for a medium‐sized laboratory and the amount for each sample as described above. The overall costs are calculated for 24 samples (maximum utilization of the CPD) as well as for a single sample to make the processes comparable. A more detailed price overview with the estimated prices and amounts used of each solvent can be found in Table S5.

**TABLE 1:** Cost overview and comparison of the different solvents with CPD drying.

Solvents	CPD(€/24 Sample)^a^, ^b^	H_2_O(€/24 Sample)^a^, ^b^	EtOH (€/24 Sample)^a^, ^b^	HMDS (€/24 Sample)^a^, ^b^	TMS (€/24 Sample)^a^, ^b^
Electricity		—	—	—	—
CO_2_	6.51	—	—	—	—
Etching‐ 1 M HCl	0.47	0.47	0.47	0.47	0.47
Washing ‐ DI‐H_2_O	0.3	0.3	0.3	0.3	0.3
Washing – 96% EtOH	4.14	—	4.14	4.14	4.14
Washing – 99% EtOH	—	—	—	3.86	3.86
Solvent	4.61	—	2.72	62.8	209.1
Maintenance	required	—	—	—	—
Purchase Value	(€10k – 32k)	—	—	—	—
Overall Cost/24 Samples	16.03	0.77	7.63	71.57	217.87
Overall Cost / Sample	0.68	0.03	0.32	2.98	9.08
Overall Cost/Sample Volume (€ cm^−3^)^a)^	3.01	3.41	1.42	13.19	40.18

^a^ Sample size 12 x 2 mm.

^b^ Prices for lab−scale procurement, ex. local taxes.

**FIGURE 7:**
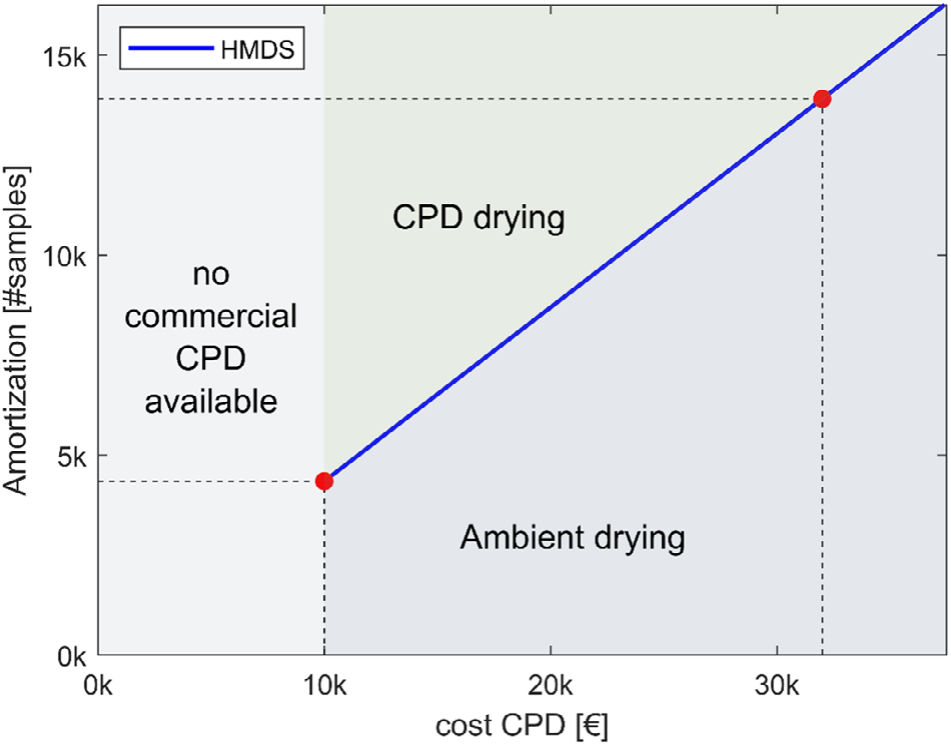
The graph shows the development of the costs for the ambient drying process as an example for HMDS. The two red dots mark exemplary prices for a favorable and a more expensive CPD, and the respective amortization of the CPD respective to the fabricated samples. The colored areas show the quantity of samples produced for which it is economically advantageous to invest in a CPD (green) or to dry the samples in the air (blue). The area below €10 000 assumes the lowest price for a CPD tool. Below this level, it is economically necessary to dry in ambient conditions.

Comparing CPD and ambient drying of FAMs, the breakeven range—depending on the CPD cost (between €10 000 and €32 000)—is between 4350 and 13 000 samples, seen in Figure 7 (for more information, see Figure S19). Therefore, for lab‐scale fabrication, at least 4350 samples must be prepared for the CPD to pay off, assuming the CPD is always operated at a full capacity of 24 samples. This also assumes the purchase of a “cheap” CPD, which may lack automatic drying, requiring direct operation for around 2 h by a trained user.

Moreover, approximately 96% of the solvent used in the final step of ambient drying from HMDS can be recovered and reused for drying, further reducing material costs and environmental impact. The recoverability of the solvent was tested using a simple condenser setup (Figure S20a), where a MXene‐FAM was placed into a flask. The FAM was immersed in HMDS and heated gently to facilitate distillation. 96% of the HMDS was captured and later used to dry another MXene‐FAM with no measurable shrinkage (Figure S20b,c). For both HMDS and TMS, the reusability is limited by the efficiency of the solvent recovery. It should be noted that HMDS hydrolyses in water, and therefore humid conditions could accelerate aging [50]. TMS, on the other hand, does not mix with water or readily decomposes. If the solvent purity is reduced over time, it can still be used for the intermediate washing steps, and at scale, techniques such as fractional distillation could be used to recover the pure solvent.

This recoverability further pushes the breakeven point upward in favor of the ambient drying using HMDS.

In addition to the cost incurred, the process time is also considered an economic factor. The following table Table 2 shows the estimated overall time for each of the different process steps.

**TABLE 2:** Time overview and comparison of the different solvents with CPD drying.

Solvents	CPD	H_2_O	EtOH	HMDS	TMS
Washing	2 h	1 h	2 h	2 h	2 h
Drying	2 h	4 h	1 h	0.5 h	0.05 h
Overall Time	4 h	5 h	3 h	2.5 h	2.05 h

If only the process time is considered, TMS leads to the fastest drying samples due to its high vapor pressure of 942 hPa (@20°C) and low boiling point of 26.6°C [51]. H_2_O, on the other hand, leads to the longest drying time due to its high surface tension. Considering deformation, process time, and price per sample, HMDS is the most suitable solvent to dry the FAMs based on the recorded data.

Furthermore, the method proposed in this paper involves fewer transfer steps, which not only simplifies handling but also enhances reliability by reducing the chances of user‐induced errors. Each additional process step inherently increases variability and the likelihood of sample damage, making our streamlined approach particularly advantageous for reproducible and scalable fabrication of highly porous materials.

At the same time, the process offers high scalability, as it is not restricted by the chamber size of CPD or freeze‐drying systems. Unlike conventional drying methods, which require costly and size‐limited vacuum or thermal treatment chambers, ambient drying conditions enable processing of larger samples or batch production without additional infrastructure investments. Scaling up CPD systems poses safety risks and practical limitations, whereas the proposed drying approach can be easily parallelized for industrial applications, with a simple hood and cold trap to recover solvent.

Using HMDS drying it was possible to dry a 11 × 11 × 0.3 cm graphene FAM. Photographs of this sample before and after drying are shown in Figure S21a,b, respectively. This far exceeds what is possible with the limited chamber size offered by typical CPD systems.

Thus, in addition to shorter processing times and reduced costs, ambient drying using HMDS enables both small‐scale precision and large‐scale manufacturability, making it a versatile and efficient alternative to conventional drying techniques.

## 4 Conclusion

The results show that ambient drying of highly porous nanomaterial structures using low surface tension solvents is a feasible alternative to critical point drying. The suitability of this approach depends on the material properties. These include mechanical and chemical properties, like interaction with the drying solvent. The method was proven to be effective for different classes of nanomaterials, conductors (MXene and Graphene), semiconductors (MoS_2_), insulators (TiO_2_), and conductive polymers (PEDOT:PSS). In terms of overall structural preservation, the best results were obtained using HMDS as the drying medium across all materials tested. Minimal deformations as low as 1.3% could be reached for MXene using HMDS and 1.3% for EG using TMS.

Beyond structural performance, the proposed process is faster, simpler, and cheaper on a lab scale as no capital investment is needed and a significant amount of solvent (96%) can be recovered during drying. Furthermore, this method has no intrinsic limitations on specimen size or quantity, making it highly versatile and suitable for upscaling. This was shown by producing a 11 × 11 × 0.3 cm EG‐FAM through ambient drying using HMDS.

We propose that this ambient drying approach may be suitable for other aerogel‐like structures and is likely not limited solely to FAMs. We are confident that this process will have wide ranging impact in the field of porous materials including energy storage or catalysis.

## Funding

DS, VN, and RA acknowledge funding by the EIC project SuperHeart (grant no. 212544, award no. 17371). RA acknowledges funding by Deutsche Forschungsgemeinschaft (DFG, German Research Foundation)—Project‐ID 434434223 – SFB 1461; Project‐ID 501386727

## Conflicts of Interest

The authors declare no conflicts of interest.

## Supporting information




**Supporting File**: smtd70661‐sup‐0001‐SuppMat.docx.

## Data Availability

The data that support the findings of this study are available from the corresponding authors upon request.
