# Supplementary material for: Low‐Cost Ambient Pressure Drying Approach for Highly Porous Nanomaterial Structures
Source: Small Methods. 2026 Apr 20;10(15):e02308. doi: 10.1002/smtd.202502308 (PMC13450365; doi:10.1002/smtd.202502308)
Supplement: Supplementary file 1 [file SMTD-10-e02308-s001.docx]

Supporting Information

Low-Cost Ambient Pressure Drying Approach for Highly Porous Nanomaterial Structures

Erik Greve*, Pia Pooker*, Jan-Ole Stern*, Rainer Adelung, Valeria Nicolosi, Dahnan Spurling*

**Table S1.** Overview of the physical properties of water, ethanol, hexamthyldisilazane and tetramethylsilane

| Solvents | Density @20°C  [g cm^-3^] | Boiling Point  [°C] | Surface Tension @ 25°C  [nM m^-1^] | Vapor Pressure @ 20°C  [hPa] | Dipole Moment  [Debye] |
| --- | --- | --- | --- | --- | --- |
| Water | 1^1^ | 100^1^ | 71.99^2^ | 33.58^3^ | 1.85 (polar)^4^ |
| Ethanol | 0.7905^1^ | 78.2^1^ | 22.39-21.55^5^ | 83.33^6^ | 1.66 (polar)^7^ |
| HMDS | 0.7741^1^ | 125^1^ | 18.2^8^ | 18.398^9^ | 0.37^10^ |
| TMS | 0.648^1^ | 26.6^1^ | 26.3^11^ | 942^1^ | 0^1^ |

**Table S2.** Atomic Composition of FAMs dried with HMDS

| FAM | Si (at. %) | O (at. %) | F (at. %) | C (at. %) | S (at. %) | Cl (at. %) | Al (at. %) | Ti (at. %) | Zn (at. %) | Mo (at. %) | Total |
| --- | --- | --- | --- | --- | --- | --- | --- | --- | --- | --- | --- |
| EG | 0.66 | 9.11 | - | 90.23 | - | - | - | - | - | - | 100 |
| PEDOT:PSS | 0.05 | 15.52 | - | 69.21 | 10.21 | - | - | - | 5.01 | - | 100 |
| MXene | 0.03 | 34.64 | 2.59 | 35.83 | - | 1.00 | 1.56 | 24.35 | - | - | 100 |
| TiO_2_ | 0.04 | 27.25 | 3.88 | 4.74 | - | - | - | 18.6 | 45.49 | - | 100 |
| MoS_2_ | 0.11 | 6.81 | - | 72.37 | 11.4 | 1.31 | - | - | 1.46 | 6.54 | 100 |

**Figure S1.** EDS Spectra of FAMs dried with HMDS.

**Figure S2.** EDS Spectra of FAMs dried using Critical Point Drying.

**Figure S3.** EDS Spectra of FAMs dried with HMDS.

**Figure S4.** SEM Image of MXene-FAM dried from H_2_O.

**Figure S5.** SEM Image of MXene-FAM dried from EtOH.

**Figure S6.** SEM Image of MXene-FAM dried from HMDS.

**Figure S7.** SEM Image of MXene-FAM dried from TMS.

**Figure S8.** SEM Image of TiO_2_ -FAM dried from H_2_O.

**Figure S9.** SEM Image of TiO_2_ -FAM dried from EtOH.

**Figure S10.** SEM Image of TiO_2_ -FAM dried from HMDS.

**Figure 11.** SEM Image of TiO_2_ FAM dried from TMS.**S**

**Figure S12.** SEM Image of MoS_2_ -FAM dried from H_2_O.

**Figure S13.** SEM Image of MoS_2_ -FAM dried from EtOH.

**Figure S14.** SEM Image of MoS_2_ - FAM dried from HMDS.

**Figure S15.** SEM Image of MoS_2_ - FAM dried from TMS.

**Figure S16.** Mechanical compression testing of EG- and PEDOT:PSS-FAMs. The dried EG-FAM shows, in comparison to the PEDOT:PSS-FAM, elastic behavior before deforming plastically.

**Figure S17.** Raman spectra of TiO_2_-FAM after critical point drying (CPD, orange) and ambient drying from TMS (blue, offset by 0.05 for clarity)^19^. Both spectra show the characteristic anatase Raman modes (E_g_, B_1g_, A_1g_) at identical peak positions.

**Table S3.** Summary of deformation and drying time values.

| Material | Solvent | Mean Deformation  [%] | Drying Time  [min] |
| --- | --- | --- | --- |
| MXene | H_2_O  EtOH  HMDS  TMS | 34.4  11.4  1.3  8.8 | 307.5  42.6  38.7  7 |
| EG | H_2_O  EtOH  HMDS  TMS | 19.4  20  4.5  1.3 | 215.6  123.5  132.9  45.5 |
| PEDOT:PSS | H_2_O  EtOH  HMDS  TMS | 61.8  25.8  14  34.8 | 210.7  51.6  42  12 |
| MoS_2_ | H_2_O  EtOH  HMDS  TMS | 11.3  20.4  7.9  10 | 255.6  69.5  43.1  66.9 |
| TiO_2_ | H_2_O  EtOH  HMDS  TMS | 32.7  18.6  18.1  24.1 | 171.2  62.8  257  81.5 |

**Table S4.** Comparison of aerogel and aerogel-like materials dried in ambient conditions. Note that the time is the total time taken post-synthesis of the precursor gel, e.g. washing/surface treatment/drying. For consistency, shrinkage is reported to the nearest %.

| **Source** | **Material** | **Density (g cm^-3^)** | **Shrinkage (%)** | **Time (h)** |
| --- | --- | --- | --- | --- |
| Nation et al. ^2^ | Biological Tissues | - | - | - |
| Wei et al.^12^ | SiO_2_ | 0.069 | 1% | 288 |
| Venkateswara Rao et al. ^13^ | SiO_2_ | 0.14 | 13% | 228 |
| Zhu et al. ^14^ | SiO_2_ | 0.10 | - | 60 |
| Hwang et al. ^15^ | SiO_2_ | 0.13 | 6% | 96 |
| Guo et al.^16^ | Graphene/carbon | 0.11 | 1% | 72 |
| Liao et al.^17^ | Polybenzoxazine | 0.322 | 6% | 24 |
| Tang et al.^18^ | Nano-cellulose | 0.050 | 10% | 8 |
| Tang et al.^18^ | Nano-cellulose-MXene | 0.052 | 21% | 8 |
| Tang et al.^18^ | Nano-cellulose-CNT | 0.064 | 20% | 8 |
| **EG-FAM (This work)** | Graphene | 0.018 | 1% | 4 |
| **PEDOT-FAM (This work)** | PEDOT:PSS | 0.022 | 5% | 4 |
| **TiO_2_-FAM (This work)** | TiO_2_ | 0.13 | 7% | 4 |
| **MX-FAM (This work)** | Ti_3_C_2_T*_x_* MXene | 0.027 | 1% | 4 |
| **MoS_2_-FAM (This work)** | MoS_2_ | 0.011 | 3% | 4 |

**Figure S18. SEM Image of EG-FAM dried with HMDS (a, c) and TMS (b, d) after 0 (a, b) and 6 Months (c, d)**

**Table S5.** Breakdown for the calculation of the cost of the drying process.

| Material | Cost ^a)^ | CPD  [ml/sample] | H_2_O  [ml/sample] | EtOH  [ml/sample] | HMDS  [ml/sample] | TMS  [ml/sample] |
| --- | --- | --- | --- | --- | --- | --- |
| HCl (1 M) | 3.94 €/L | 5 | 5 | 5 | 5 | 5 |
| DI-H_2_O | 0.5 €/L | 25 | 25 | 25 | 25 | 25 |
| EtOH (96%) | 11.1 €/L | 25 | - | 25 | 25 | 25 |
| EtOH (99%) | 18.9 €/L | 6+ (17+ 83) (/24 samples) ^b)^ | - | 6 | 8,5 | 8,5 |
| HMDS | 308 €/L | - | - | - | 8,5 | - |
| TMS | 1025 €/L | - | - | - | - | 8,5 |
| CO_2_ | 455€ per Bottle | 59.5 | - | - | - | - |

^a) Prices for lab-scale procurement, ex. Tax^
^b) 6 ml for washing each sample, 17 ml for preparation and 83 ml for filling the CPD with 24 samples^

**Figure S19.** Cost of HMDS and CPD drying.

The slope of Figure 13was calculated with the following formula:

(13,913 samples - 4,348 samples)/(€32,000-€10,000)

**Figure S19.** Schematic of the distillation setup used for the recovery of HMDS, Images of an MXene FAM dried in the recovered HMDS before (b) and after (c) drying.

**Figure S20.** Image of an 11x11x0.3 cm graphene-FAM dried from HMDS (a) wet and (b) dry

**References**

1.	Lide, D. R. *CRC Handbook of Chemistry and Physics, 85th Edition*. (CRC Press, 2004).

2.	Nation, J. L. A New Method Using Hexamethyldisilazane for Preparation of Soft Insect Tissues for Scanning Electron Microscopy. *Stain Technology* **58**, 347–351 (1983).

3.	Bridgeman, O. C. & Aldrich, E. W. Vapor Pressure Tables for Water. *J. Heat Transfer* **86**, 279–286 (1964).

4.	Mortimer, C. E. & Müller, U. *Chemie: das Basiswissen der Chemie ; 126 Tabellen*. (Georg Thieme Verlag, 2007).

5.	https://srd.nist.gov/JPCRD/jpcrd13.pdf. https://srd.nist.gov/JPCRD/jpcrd13.pdf.

6.	Ambrose, D. & Sprake, C. H. S. Thermodynamic properties of organic oxygen compounds XXV. Vapour pressures and normal boiling temperatures of aliphatic alcohols. *The Journal of Chemical Thermodynamics* **2**, 631–645 (1970).

7.	Informatics, N. O. of D. and. Ethanol. https://webbook.nist.gov/cgi/cbook.cgi?ID=64-17-5&Type=IR-SPEC&Index=QUANT-IR,9.

8.	Myers, R. S. & Clever, H. L. Surface tension of octamethylcyclotetrasiloxane and hexamethyldisilazane and their solutions with carbon tetrachloride and n-hexadecane. *J. Chem. Eng. Data* **14**, 161–164 (1969).

9.	PubChem. Hexamethyldisilazane. https://pubchem.ncbi.nlm.nih.gov/compound/13838.

10.	1,1,1,3,3,3-HEXAMETHYLDISILAZANE, 98% |. https://www.gelest.com/product/SIH6110.0/.

11.	Dey, S. 12. Sudip Dey, TS. Basu Baul, B Roy & D Dey (1989): A new rapid method of air-drying for scanning electron microscopy using tetra-methyl silane. J. Microscopy. (Oxford). 156: 259-261. *J.Microscopy* **156**, 259–261 (1989).

12.	Wei, T., Chang, T., Lu, S. & Chang, Y. Preparation of Monolithic Silica Aerogel of Low Thermal Conductivity by Ambient Pressure Drying. *Journal of the American Ceramic Society* **90**, 2003–2007 (2007).

13.	Venkateswara Rao, A., Nilsen, E. & Einarsrud, M.-A. Effect of precursors, methylation agents and solvents on the physicochemical properties of silica aerogels prepared by atmospheric pressure drying method. *Journal of Non-Crystalline Solids* **296**, 165–171 (2001).

14.	Zhu, L. *et al.* Preparation of Silica Aerogels by Ambient Pressure Drying without Causing Equipment Corrosion. *Molecules* **23**, 1935 (2018).

15.	Hwang, S.-W., Jung, H.-H., Hyun, S.-H. & Ahn, Y.-S. Effective preparation of crack-free silica aerogels via ambient drying. *J Sol-Gel Sci Technol* **41**, 139–146 (2007).

16.	Guo, K. *et al.* Low-density graphene/carbon composite aerogels prepared at ambient pressure with high mechanical strength and low thermal conductivity. *RSC Adv.* **5**, 5197–5204 (2015).

17.	Liao, Y. *et al.* Ambient drying to fabricate polybenzoxazine aerogels for thermal insulation in aerospace. *Materials Today Nano* **28**, 100517 (2024).

18.	Tang, C., Yang, J., Liu, J., Mao, Y. & Fu, S. A scalable, efficient, and facile ambient drying approach for preparing low shrinkage, compressive, and superporous nano-cellulose-based aerogels *via* a physicochemical crosslinking strategy. *Green Chem.* **27**, 804–814 (2025).

19. Ohsaka, T., Izumi, F. & Fujiki, Y. Raman Spectrum of Anatase, TiO_2_. *J. Raman Spectrosc.* **7**, 321–324 (1978).
